# Searching for online information on the fit of children’s footwear during the COVID-19 pandemic: an analysis of Google Trends data

**DOI:** 10.1186/s13047-022-00598-5

**Published:** 2023-01-09

**Authors:** Carina Price, Stewart Morrison, Michael Haley, Christopher Nester, Anita Williams

**Affiliations:** 1 Human Movement and Rehabilitation Research Group, University of Salford, Brian Blatchford Building, Frederick Road Campus, Salford, England; 2 School of Life Course and Population Sciences, Faculty of Life Sciences and Medicine, King’s College, London, England; 3 School of Health and Society, University of Salford, Brian Blatchford Building, Frederick Road Campus, Salford, England

**Keywords:** Footwear, Children, Fit, Online purchase, COVID, Health advice

## Abstract

**Background:**

Selecting footwear with appropriate fit in children is challenging due the changes with foot size and dimensions which occur throughout childhood. Access to appropriate footwear is important but recent challenges with the COVID-19 pandemic resulted in closure of retail stores for prolonged periods where parents/carers could not physically purchase footwear for their children and the footwear industry suffered disruption to their supply chain, and falls in retail sales. Simultaneously increased use of social media platforms for health information seeking throughout the pandemic have been documented. This likely would have included parents/carers seeking information online to support footwear purchases for their children. The primary aim of this work was to explore how searches for online fitting information for children changed throughout the COVID-19 pandemic lockdown periods. A secondary aim was to identify how searches were influenced by footwear style.

**Methods:**

We employed Google Trends to obtain search engine traffic related to footwear fitting information for children. We collected data spanning the three years pre, during and post the main national lockdown for three eight-week windows: (1) first eight weeks of the U.K. national lockdown; (2) the first eight weeks of the calendaryear; (3) the eight weeks leading up to children going back-to-school for the new academic year in the U.K. The search terms reflected parents/carers searching for footwear fit information relating to children and were grouped by style of footwear: children, infants, babies and toddlers as well as school shoes.

**Results:**

We identified increased searching for footwear fit information for children during the pandemic, which reduced following post pandemic in all except the searches which related to school shoes. We saw reductions in searching related to fit of school shoes as schools closed indefinitely and an increase in online searches with the pandemic. This was also maintained post-pandemic despite shops reopening, suggesting that some of these changes in information reflect new consumer behaviours which may continue.

**Conclusions:**

Increased searches for online resources regarding footwear fit highlights the importance of ensuring high quality accessible online information on footwear fit is available to support those buying footwear for their children.

**Supplementary Information:**

The online version contains supplementary material available at 10.1186/s13047-022-00598-5.

## Background

Footwear fit is key to ensure that footwear supports foot health, particularly in children [1, 2]. The feet are important for supporting gross motor development and simultaneously are changing in shape and dimensions at variable rates throughout childhood. During infancy we can anticipate foot length increasing by an average 2 mm per month, but by school age this declines to less than 1 mm [3, 4]. Achieving appropriate footwear fit in children is challenging, with reports highlighting that more than half of children wear footwear that doesn’t fit appropriately [5, 6]. Some of these challenges likely relate to the inconsistent and rapid growth rates across childhood. Other challenges may be related to footwear preference in children and the importance of social fit of footwear, which again differs with age and becoming more important in children of a school age [7]. Assessing footwear fit in children can include assessment of foot length and an adequate toe allowance [3], requirements which have traditionally been assessed in a retail store with a range of shoes to try on.

The COVID-19 pandemic led to the closure of non-essential retail stores on 23rd March 2020 in the U.K. [8]. Non-essential retail reopened in June, but further regional closures followed throughout the U.K. for the rest of 2020, determined by local COVID-19 rates and hospital admissions [8]. Further large-scale closure of retail occurred with national ‘lockdowns’ 5th November to 2nd December 2020 and 5th January 2021 to 12 April 2021 [9]. These restrictions led to a considerable drop in customers entering retail stores compared to the year prior, approximately 80% in the first week of the first national lockdown [8]. Alongside retail closures measures were implemented such as furloughing staff, job losses, school closures and a mandate to work at home. These had substantial impact on the economy and the total volume of retail sales fell 1.9% from the previous year [8]. Closing retail outlets increased online shopping, with online sales recording a high of 34% of all retail sales in May 2020 [8]. 28% of British adults reported doing more online shopping for shoes and/or clothing in this time compared to pre-pandemic [10]. Even post- the lockdowns and associated retail closures, shops continued to require, then later, advise mask-wearing until spring 2022.

Before the pandemic there was an emerging pattern of growth in the online sales of footwear, with increased accessibility driven by widespread internet access. This growth led to an increasing revenue of 6.3–9.2% per annum predicted in 2018 [11]. The COVID-19 pandemic led to a concurrent drop in sales [8], but a movement towards online shopping. This shift meant that e-commerce provision, footwear fitting information and technology improvements were rapidly implemented. Technologies relating to smart phones and e-commerce had already encouraged more widespread adoption of online ordering and purchasing [12] and it is likely that further improvement may support new and sustained consumer habits [12]. There is evidence that the COVID-19 pandemic has had a longer-term influence on the retail sector [13], changing consumer behaviours [14].

Purchasing footwear online represents a substantial shift in behaviour with consumers not being able to see shoes, try them on, or discuss appropriateness with retail staff or professionals, some of whom are trained in shoe fitting. Online purchases rely on fitting information and advice or tools relating to measure and fit. Our previous work identified concerns from professionals relating to the validity of some of these online resources for determining and checking shoe size and fit in children [15]. This invites exploration of how the COVID-19 pandemic influenced parents searching for online information relating to footwear fit when traditional routes to assure footwear fit became more limited due to retail closures. Inspired by Telfer et al., [16], we employed Google Trends to explore how searches for online fitting information for children changed throughout the COVID-19 pandemic lockdown periods. We also aimed to identify how searches were influenced by footwear style.

## Method

Data was searched and extracted from Google Trends. Google Trends is a freely available tool which enables the user to search internet search data from Google for defined terms and defined periods. The data is anonymised, categorised and aggregated to display interest in particular topics from geographical regions. Within Google Trends, data is normalised to 0- based on time and location so that comparisons can be made between different search dates and regions where search volumes differ [17]. Our searches were undertaken capturing weekly search data for three years starting on 23rd March 2019 (and ending on 23rd March 2022) and undertaken in the U.K. only. These years were defined pre- (23rd March 2019- 21st March 2020), pandemic (22nd March 2020- 20th March 2021) and post- (21st March 2021- 19th March 2022). Search terms were related to footwear information and based on those utilised previously for our online review (Table 1) [15]. These keywords related to parents seeking footwear fit and measurement information for children’s shoe styles.

**Table 1:** Search terms for Google Trends searches to quantify parents seeking online information about children’s footwear fit and sizing relating to online purchases

**Child term**	**Foot or Footwear term**	**Fit term**
Child/children	Shoe (s)	Size
Infant (s, ‘s)	Footwear	Sizing
Baby/babies	Foot	Size chart
Toddler (s, ‘s)		Fit
**Stage term**		Fitting
First		Information
School		Advice
Pre-walker		Help
Cruising		Guide
**Additional terms**		Tools
Measure [child term] feet		Measure
[child term] foot measure		Scale

The normalised (0-100) weekly search volumes spanning the three-year period from Google Trends were downloaded in .csv format. Some search terms did not report enough data to provide a result, so do not contribute to the data. Official lockdown dates (as reported in the introduction) and national (English) school term dates were recorded for reference.

Individual search terms were grouped into pre-determined categories representing different styles of footwear across childhood: baby, infant, toddler, child and school. These categories were determined with consensus from the authors after considering different footwear requirements at different ages and are based on the noun used in the main search string. Median values were calculated for all searches within each style. Statistical comparison compared median values across eight-week windows (pre-, pandemic and post-) from 23rd March 2019–19th March 2022. This used Friedman and related samples Wilcoxon Signed Rank Tests using IBM SPSS (Statistical package for the social sciences) V27. Three comparisons were made:

1. The immediate impact of COVID-19: This compared the eight weeks of initial lockdown in the U.K.: 3rd week of March to 2nd week of May 2020 (pandemic) compared to the same time window pre-pandemic (2019) as a baseline. Comparisons were made using Wilcoxon Signed Rank Test.
2. The lasting influence of COVID-19: This compared the eight weeks from 4th week of January to 3rd week of March for all three years (pre-, pandemic and post-). This period was one where retail was open (pre-), closed (pandemic) and open (post-pandemic). Comparisons were made using Freidman test then post-hoc related samples Wilcoxon Signed Rank Tests to determine differences.
3. The influence of COVID-19 on school shoes: This compared the eight weeks (3rd week of July to 2nd week of September) leading up to the return of schools for the new academic year for all three years (pre-, pandemic and post-). This date in the pandemic year was the one in which schools re-opened in the U.K. following closing in March 2019 for the first national lockdown. Comparisons were made using Freidman test then post-hoc related samples Wilcoxon Signed Rank Tests to determine differences.

## Results

**Fig. 1:**
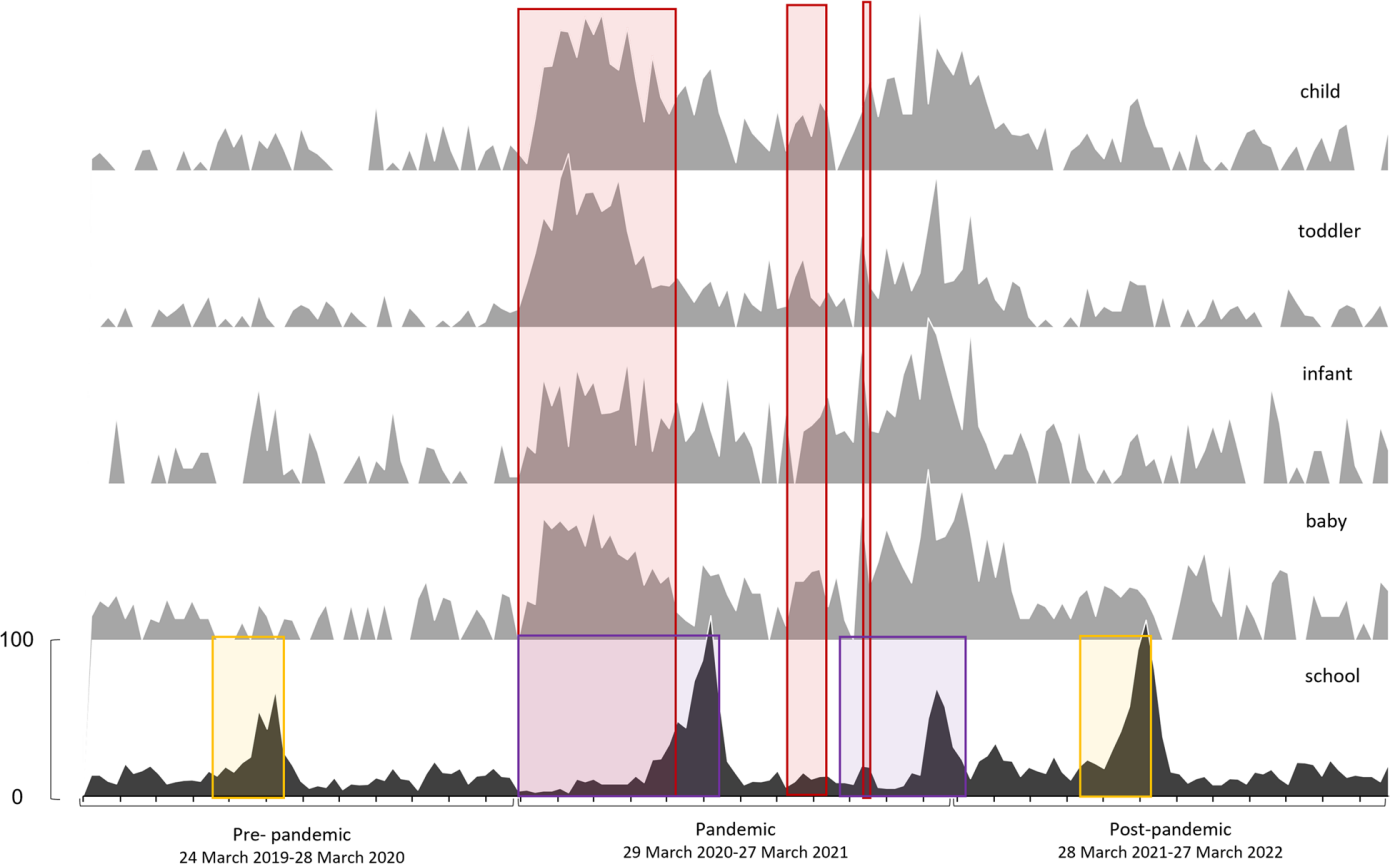
Median number of searches (normalised 0-100) for all footwear styles spanning the three years of data included in the Google Trends search (pre-, pandemic and post-) displaying relevant dates. X-axis tick parks represent months, red marked areas represent the periods of retail closure, yellow marked areas represent the standard school closure in England and purple marked areas additional school closures due to the pandemic

**Fig. 2:**
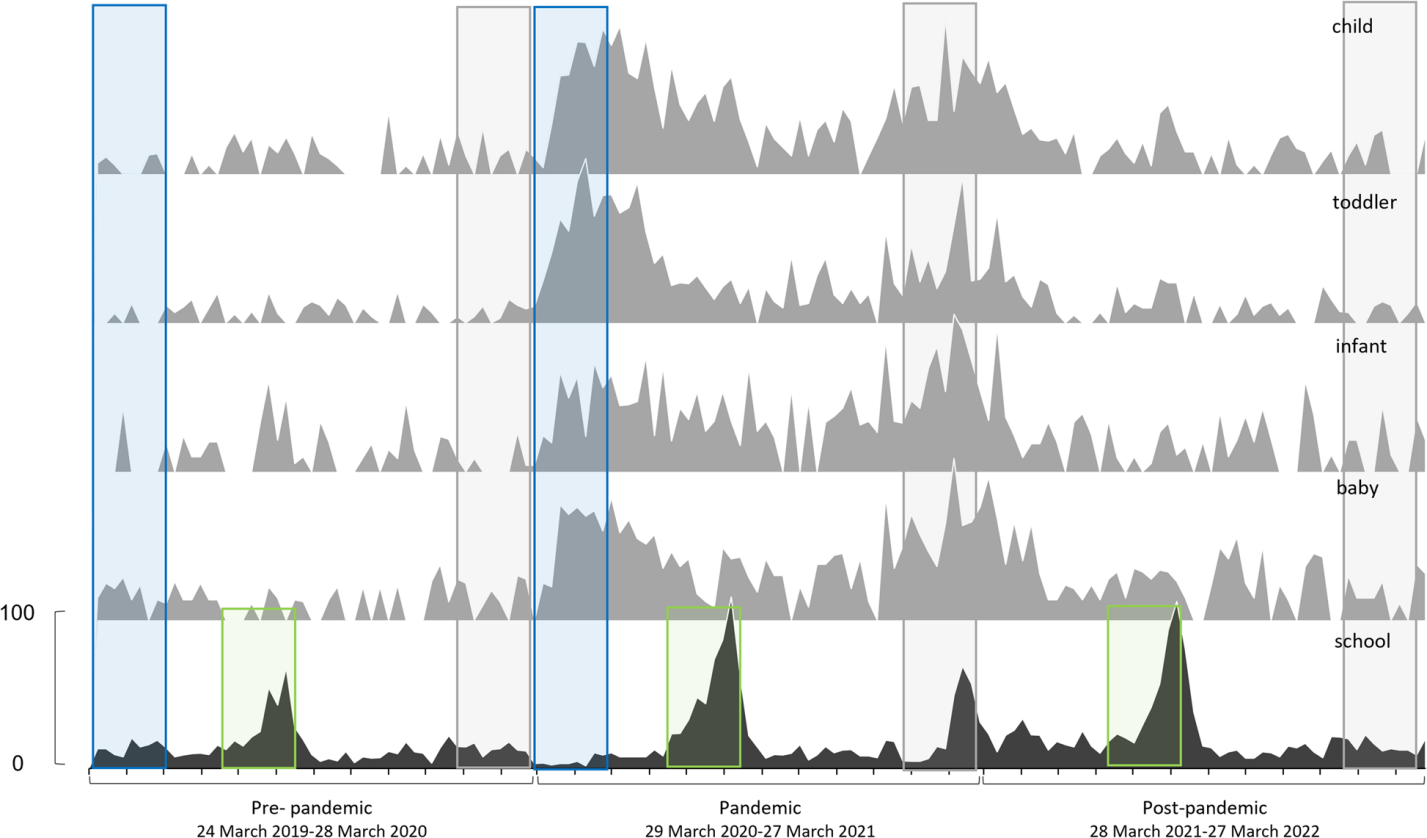
Median number of searches (normalised 0-100) for all footwear styles spanning the three years of data included in the Google Trends search (pre-, pandemic and post-) displaying the comparisons undertaken. (1) blue marked areas compare the immediate impact of COVID-19; (2) grey marked areas compare the lasting influence of COVID-19; (3) green marked areas compare the influence of COVID-19 on school shoes. X-axis tick parks represent months

**Fig. 3:**
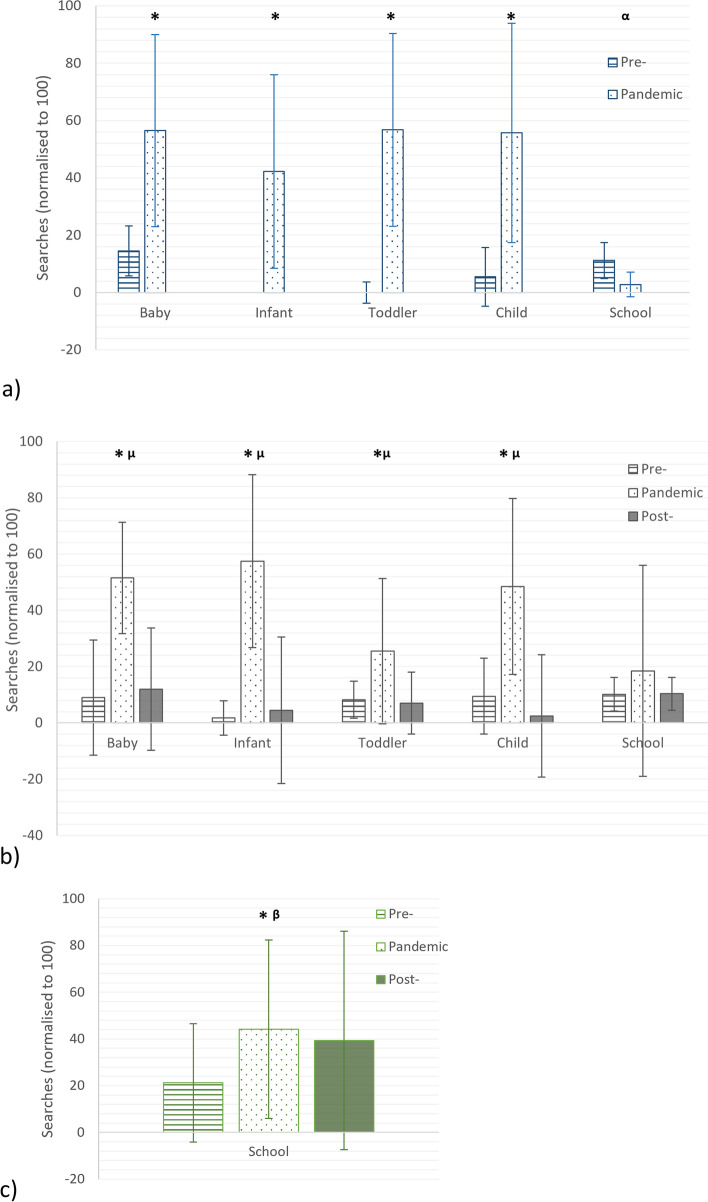
Median (+/- IQR) normalised searches for each shoe style for eight-week windows (**a**) the immediate impact of COVID-19 (**b**) the lasting influence of COVID-19 (**c**) the influence of COVID-19 on school shoes. Where * = pre- < pandemic, ^α^ = pandemic < pre-, ^µ^ = post- < pandemic, ^β^ = pre- < post with *p*  ≤ .05

Of the 312 searches undertaken in Google Trends, those with the fit terms ‘sizing’, ‘advice’, ‘help’, ‘tool’ and ‘scale’ did not return enough data to be included. Similarly searches including ‘footwear’ as a footwear term and ‘kid*’ as a child term also did not have enough search data to be reported by Google Trends. Our total number of searches included was 44 and this was a minimum of five for each footwear style. The date outcomes form Google Trends for each footwear style and the key dates for national retail closures and school term dates (Fig. 1) and key outcome comparisons are presented (Fig. 2). The equivalent median data for the styles for each pandemic year alongside statistical outcomes are presented in Fig. 3.

The immediate influence of COVID-19 was quantified in the eight weeks of initial lockdown (Fig. 3a). Searches were higher during the pandemic than the year pre-pandemic for baby (median 56.5 IQR 33.5 v 14.5 IQR 8.8, *p* = .017), infant (median 42.3 IQR 33.8 v 0 IQR 0, *p* = .012), toddler (median 56.8 IQR 33.7 v 0 IQR 3, *p* = .012) and child (median 55.8 IQR 38.3 v 5.5 IQR 10.3, *p* = .017) styles (Fig. 3a). However, searches related to school styles were reversed (median 11.2 IQR 6.3 v 2.8 IQR 4.3, *p* = .012), likely due to schools being closed for an undetermined amount of time during the pandemic year (Fig. 3a). The on-going influence of COVID-19 was explored by comparing the year pre- pandemic, the year of the pandemic and the year post-pandemic during the first eight weeks of each year, when retail was open pre- and post-pandemic (Fig. 3b). The year post-pandemic, searches were significantly lower in volume than pandemic year, back to pre- pandemic levels in baby (*p* = .002), infant (*p* = .001), toddler (*p* = .002) and child (*p* = .002) styles. For school styles however, searches remained elevated post-pandemic and did not return to pre-pandemic levels (*p* = .368) (Fig. 3b). We also explored the eight weeks of search data leading up to children returning to school (second week of September 2019, 2020 & 2021). Data from the pandemic year (median 44.2 IQR 38.2, *p* = .006) and the post-pandemic (median 39.3 IQR 46.8, *p* = .001) year showed elevated levels of search terms compared to the pre-pandemic (median 21.2 IQR 25.3) year (Fig. 3c).

## Discussion

The data identifies that there was greater reliance on the internet for information related to children’s footwear fit during the pandemic and periods of retail closure. Searches increased at lockdown during the pandemic and then reduced to pre- pandemic levels following the re-opening of retail in all styles except school shoes. For school shoes, we identified that pre-, pandemic and post-pandemic searches didn’t differ at the start of the year. Also, that pre-pandemic searches were lower than pandemic and post pandemic searches during the 8 weeks prior to the new school year starting.

We are unable to confirm how purchasing behaviours have been influenced, but our data shows that online information seeking related to the fit of school shoes was maintained after retail stores re-opened. This suggests transferring consumers to online information seeking may have longevity in the school shoe market and reinforces the importance of footwear companies being able to support high quality online information, particularly around school footwear during the period before the new school year starts. The return to normal levels of searching for other styles reflects different consumer behaviours for footwear of different styles. This may be due to the cost of the footwear, parent confidence relating to self-determining fit, the role of the footwear or a complex interplay of many factors (including levels of emotion surrounding and meaning of the shoe being purchased) [18], however we cannot determine this from our current data set. The differing results for school shoes may also reflect pre-pandemic levels of online information searching already being higher than other footwear styles (see pre-pandemic year on Figs. 2 and 3). Higher levels of information searching, driven by increased internet availability and potentially the COVID-19 pandemic, escalates the importance of valid and credible online information related to footwear fit in children. Our prior research questioned the validity of some of this online information [15]. Of the 15 most accessible resources online 12 scored less than five out of 10 for the extent to which the professional panel deemed measures to be valid for parents to undertake to measure their children’s feet and assess their footwear fit. However, this work was undertaken in 2019 and since the COVID-19 pandemic there have been updates to e-commerce platforms from numerous footwear companies, some of which include 3D applications to measure feet using smartphones. These upgrades would likely improve the quality and validity of information being delivered to parents to select footwear with appropriate fit at home.

Aside from store closures, other factors likely influenced footwear purchasing behaviour, and parents seeking information online about children’s shoes. Consumption is habitual and changes in routine and activities during the pandemic could have altered behaviour [19]. Other factors such as safety concerns with a fear that COVID-19 could be spread on products, may have also mediated purchase of footwear [20]. From an industry perspective, the supply-chains and exports were also substantially affected [21–23], which may have limited the availability of some footwear styles in some markets. The influence on the economy was substantial, reducing household incomes and discretionary purchases, which may have reduced purchases of footwear. Children’s footwear, however, may be deemed an essential spend by a parent due to foot growth and therefore been prioritised, particularly in younger children with higher growth rates. Contrasting this, for school shoe styles, school closures would have meant no requirement for uniform specific shoes therefore potentially reducing related searches. These contrasts may result in differing patterns of online searching across footwear styles. Ultimately these factors would have all interacted to alter search behaviour of parents looking to search for online fit information for their children.

There are limitations associated with this data, which is based on Google searches alone, as opposed to other search engines, and individuals searching for fit advice and not direct footwear purchases. Results are specific to the U.K. due to the search restrictions imposed and relevance of dates chosen. We would also like to acknowledge that not everyone has been able to return to shopping in retail stores due to health status.

## Conclusion

There was greater reliance on Google searches for information related to children’s footwear fit during the periods of retail closures in the COVID-19 pandemic. Searches relating to most children’s styles increased at lockdown and then reduced to pre- pandemic levels following the re-opening of retail. Searches for school shoes demonstrated a different pattern where searching volumes remained elevated even when retail stores had re-opened. Despite the limitations of this study, the data supports that there is an ongoing reliance on the internet for information on footwear and fitting in children searching for online information on footwear and fitting. Hence, health professionals and the retail sector have roles to play in ensuring high quality accessible online information on footwear fit is available as well as how to best support parents with the transition to online information seeking for footwear fitting advice. This will support those buying footwear for their children to purchase footwear of an adequate fit. Currently it seems this is particularly important for school shoes.

## Supplementary Information

**Additional file 1.**

## Data Availability

The datasets used and/or analysed during the current study are publicly available using the data collection tool reported in the methods. They are included in the published article within the figure and are also available from the corresponding author on reasonable request.
