# Supplementary material for: Searching for online information on the fit of children’s footwear during the COVID-19 pandemic: an analysis of Google Trends data
Source: J Foot Ankle Res. 2023 Jan 9;16:1. doi: 10.1186/s13047-022-00598-5 (PMC9826755; doi:10.1186/s13047-022-00598-5)
Supplement: Supplementary file 1 — Additional file 1. [file 13047_2022_598_MOESM1_ESM.docx]

20^th^ September 2022

Dear Editor,

Please consider our amendments to our paper for submission for publication within JFAR:

*Searching for online information on the fit of children’s footwear during the COVID-19 pandemic: an analysis of Google Trend data*

We believe this to be a timely topic and within scope of your readership from a clinical and academic perspective.

We would like to highlight that the main revision we have undertaken in the review process is to alter the article type to a Research Paper as opposed to a commentary. Due to the requests from the reviewers it was clear that we required more word count to discuss the background, methods and results. The paper is also research, therefore is within scope for this type of submission.

Thanks again in advance for your consideration,

Dr Carina Price

University of Salford

Dr Carina Price

Dr Stewart Morrison

Michael Haley

Prof Christopher Nester

Dr Anita Williams
